# Clinical Validation of a Volumetric Absorptive Micro-Sampling Device for Pharmacokinetic Studies With Tranexamic Acid

**DOI:** 10.3389/fphar.2021.764379

**Published:** 2021-11-23

**Authors:** Stanislas Grassin-Delyle, Elodie Lamy, Michaela Semeraro, Iléana Runge, Jean-Marc Treluyer, Raoul Mansukhani, Monica Arribas, Ian Roberts, Haleema Shakur-Still

**Affiliations:** ^1^ Département de Biotechnologie de la Santé, Université Paris-Saclay, UVSQ, INSERM, Infection et Inflammation, Montigny le Bretonneux, France; ^2^ Département des Maladies des Voies Respiratoires, Hôpital Foch, Suresnes, France; ^3^ Centre d’Investigation Clinique P1419, INSERM, Hôpital Cochin-Necker, Université de Paris, Paris, France; ^4^ Unité de Recherche Clinique, Hôpital Cochin-Necker, Université de Paris, Paris, France; ^5^ Clinical Trials Unit, London School of Hygiene & Tropical Medicine, London, United Kingdom

**Keywords:** tranexamic acid, volumetric absorptive micro-sampling, pharmacokinetics, whole blood, capillary blood

## Abstract

We assessed the accuracy of tranexamic acid (TXA) concentrations measured in capillary whole blood using volumetric absorptive micro-sampling (VAMS) devices. Paired venous and VAMS capillary blood samples were collected from 15 healthy volunteers participating in a pharmacokinetic study of alternative routes (oral, IM and IV) of administering TXA. To assess accuracy across a range of concentrations, blood was drawn at different times after TXA administration. We measured TXA concentrations in plasma, whole blood from samples collected by venepuncture and whole blood from venous and capillary samples collected using VAMS devices. TXA was measured using a validated high sensitivity liquid chromatography - mass spectrometry method. We used Bland-Altman plots to describe the agreement between the TXA concentrations obtained with the different methods. In the 42 matched samples, the mean plasma TXA concentration was 14.0 mg/L (range 2.6–36.5 mg/L) whereas the corresponding whole blood TXA concentration was 7.7 mg/L (range 1.6–17.5 mg/L). When comparing TXA concentrations in VAMS samples of venous and capillary whole blood, the average bias was 0.07 mg/L (lower and upper 95% limits of agreement: −2.1 and 2.2 mg/L respectively). When comparing TXA concentrations in venous whole blood and VAMS capillary whole blood, the average bias was 0.7 mg/L (limits of agreement: −2.7 and 4.0 mg/L). Volumetric absorptive micro-sampling devices are sufficiently accurate for use in pharmacokinetic studies of tranexamic acid treatment in the range of plasma concentrations relevant for the assessment of fibrinolysis inhibition.

## Introduction

Although tranexamic acid (TXA) has been marketed for the prevention of bleeding since the 1960s and has been used in a range of surgical and out-of-hospital indications, the dosing regimens used are mostly empirical. High quality clinical trials with sufficient power to support efficacy in acute severe bleeding are relatively recent, as are the pharmacokinetic studies (Collaborators, 2019; Collaborators et al., 2010; Woman Trial Collaborators, 2017; Grassin-Delyle et al., 2021a; Grassin-Delyle et al., 2021b; Grassin-Delyle et al., 2018; Grassin-Delyle et al., 2013a). Timely TXA treatment, ideally within an hour of bleeding onset, has been shown to be essential for maximal efficacy in acute severe bleeding and so effective TXA blood concentrations must be achieved rapidly (Collaborators et al., 2011; Gayet-Ageron et al., 2018). For this, knowledge of the pharmacokinetics of TXA in each population that could benefit from this treatment is fundamental. Early intravenous (IV) administration of TXA reduces deaths from post-partum haemorrhage (Woman Trial Collaborators, 2017). To facilitate the treatment of women who give birth in community settings, the WHO recommended that “research into other routes of administration is a priority” (WHO, 2017). In response, we initiated a programme of pharmacokinetic (PK) research on alternative routes of TXA administration. Finding new routes of TXA administration is of particular interest in low- and middle-income countries and several studies have been initiated in these settings. The availability of qualified staff for venipuncture, as well as the necessary laboratory equipment for pre-analytical processing and storage of blood samples, is a prerequisite for pharmacokinetic studies. Because capillary blood sampling is simpler, less invasive and usually less painful than venepuncture (Abu-Rabie et al., 2019; Koster et al., 2019), we tested the use of a volumetric absorptive micro-sampling (VAMS) device for TXA quantification. Here we report the accuracy of TXA concentrations in blood collected using theses VAMS devices.

## Methods

As part of a randomised, cross-over PK study of alternative routes of TXA administration in healthy volunteers conducted at the Clinical Investigation Centre of Necker Hospital in Paris, we assessed the accuracy of TXA concentrations collected using volumetric absorptive micro-sampling devices. The study was approved by the London School of Hygiene & Tropical Medicine ethics committee (16286) and the Comité de Protection des Personnes Île de France III (2019-000285-38) and registered in the EudraCT (2019-000285-38) and ClinicalTrials.gov (NCT03777488) databases. The study methods are described in detail elsewhere (Grassin-Delyle et al., 2021a). Briefly, adult volunteers (non-pregnant women and men) aged between 18 and 45 years received TXA by three routes (1 g intravenous, 1 g intramuscular, 2 g oral) on three separate days with a minimum washout period of 48 h between each treatment. After each administration (T0), we took paired venous blood samples (0.5 ml venous blood in a sodium heparin tube) and duplicate capillary blood samples using the 10 µL Mitra^®^ VAMS device (Neoteryx, Torrance, CA, United States) at one of the following timepoints: T0 + 5 min (IV route only), T0 + 30 min, T0 + 1 h, T0 + 2 h, T0 + 3 h, T0 + 4 h, T0 + 5 h, T0 + 6 h, T0 + 8 h (IM and PO routes only), T0 + 24 h. Sampling with the VAMS device was performed according to manufacturer’s instructions. Once all clinical samples were obtained, they were sent and processed in batch in the laboratory.

We measured TXA concentrations in plasma and whole blood in samples collected by venepuncture. We also measured TXA concentrations in VAMS devices soaked in the whole blood collected by venepuncture and in capillary whole blood samples collected using the VAMS device. All TXA measurements were made using liquid chromatography - mass spectrometry methods (Fabresse et al., 2017; Lamy et al., 2020). For plasma, the lower limit of quantification is 0.1 mg/L with precision in the range 1.2–3.0% and an accuracy of between 88.4 and 96.6% across the range 0.1–1,000.0 mg/L. For other samples, the lower limit of quantification is 0.1 mg/L with and a precision <12.6% and an accuracy between 85.2 and 112.8% across the range 0.1–1,000.0 mg/L.

We used Bland-Altman plots to describe the agreement between the TXA concentrations obtained using the different sampling methods (Bland and Altman, 1999). First, to assess the impact of capillary sampling, we compared TXA concentrations in VAMS devices with venous and capillary blood. Second, to assess the impact of using the VAMS device, we compared TXA concentrations in venous whole blood and VAMS capillary blood. Finally, to assess whether capillary samples can be used to estimate plasma TXA concentrations, we examined the association between plasma TXA levels and those estimated from VAMS capillary blood samples using Equation 1 below, as described in our previous paper (Grassin-Delyle et al., 2021a):

$$C_{plasma}=C_{whole\;blood}\times \frac{1}{1-Ht}$$ (1)

## Results

**FIGURE 1:**
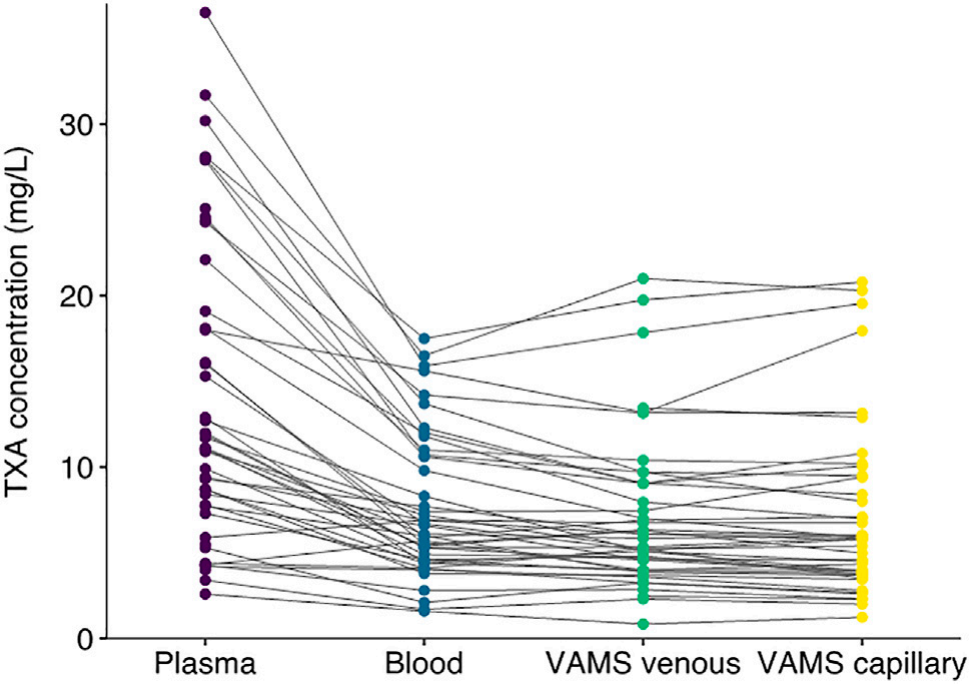
Tranexamic acid concentration in mg/L measured in plasma, whole blood, VAMS venous and VAMS capillary samples (*n* = 42).

**FIGURE 2:**
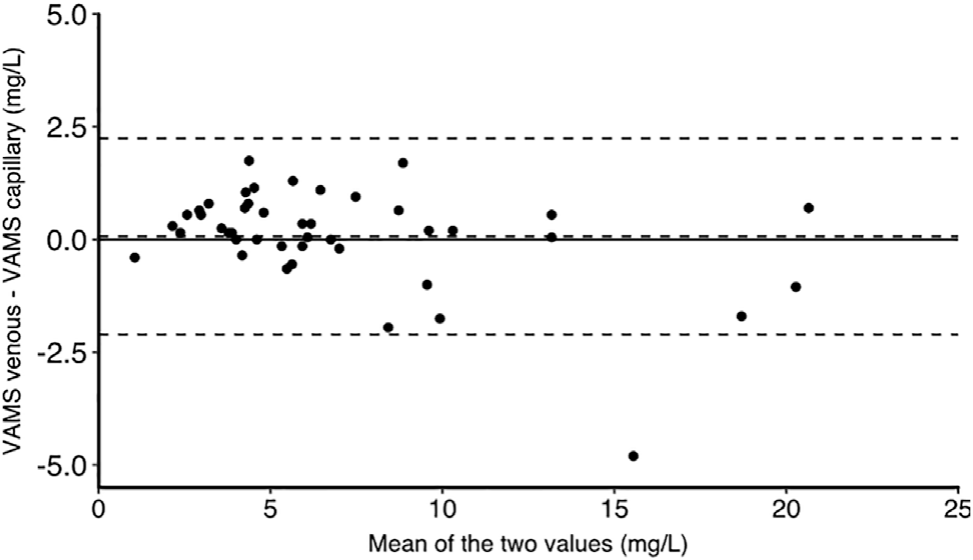
Bland Altman graph for tranexamic acid concentration in mg/L measured in VAMS samples of venous and capillary blood. The bias, lower and upper limits of agreement are at 0.07, −2.1 and 2.2 mg/L respectively (*n* = 44).

**FIGURE 3:**
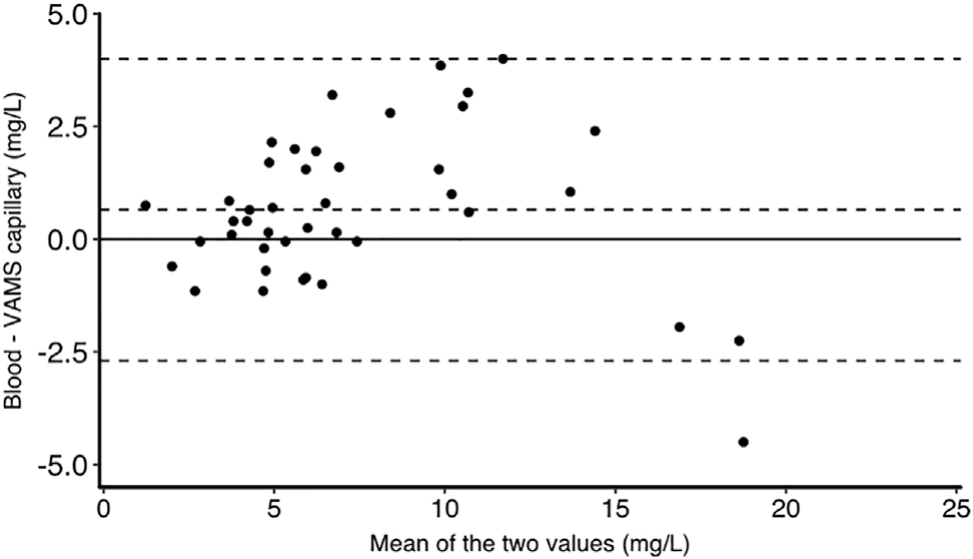
Bland Altman graph for tranexamic acid concentration in mg/L measured in venous whole blood and VAMS capillary blood. The bias, lower and upper limits of agreement are at 0.7, −2.7 and 4.0 mg/L respectively (*n* = 42).

**FIGURE 4:**
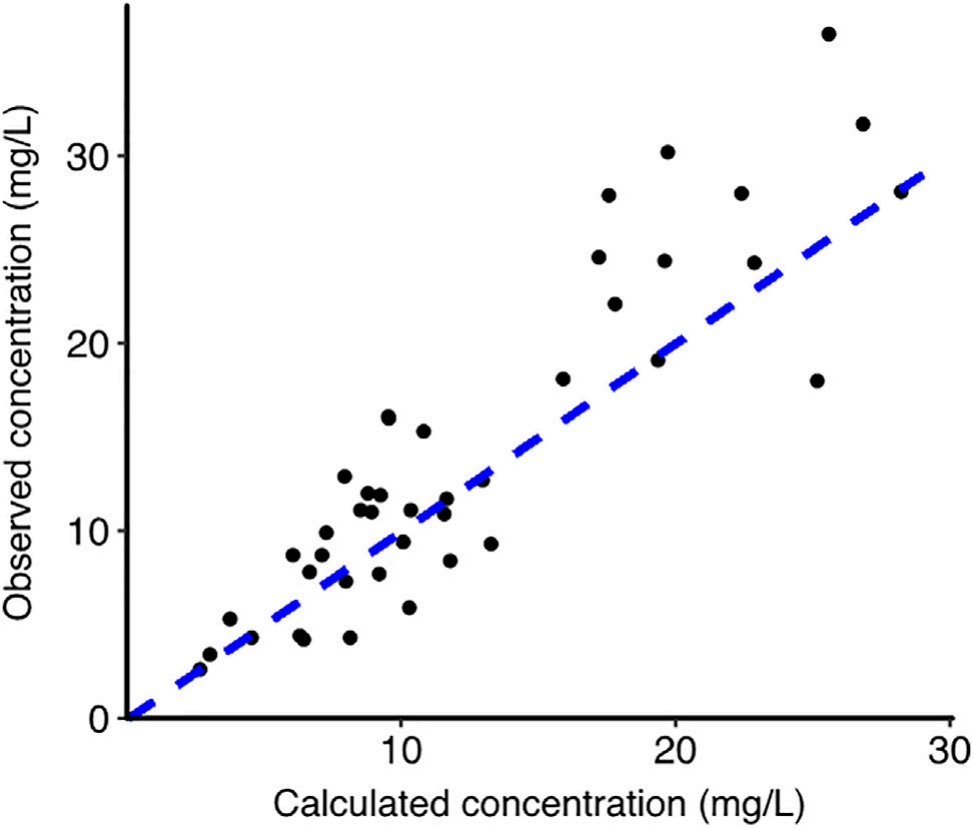
Scatter plot for tranexamic acid concentration in mg/L measured in plasma versus the calculated plasma concentration estimated from VAMS capillary blood samples (*n* = 42). The blue dotted line represents the line of identity (*y = x*).

The characteristics of the study population have been reported previously (Grassin-Delyle et al., 2021a). There were 11 women and four men. The median age was 25 years and the median bodyweight was 64.2 kg. We obtained 42 matched venous plasma, venous whole blood, venous whole blood on VAMS devices and capillary VAMS samples. There were three missing values for venous whole blood samples and one missing venous VAMS sample. The TXA concentrations in all samples are shown in Figure 1. TXA concentrations in plasma were higher than in whole blood. The mean (SD) plasma TXA concentration was 14.0 (8.9) mg/L whereas the mean whole blood TXA concentration was 7.7 (4.3) mg/L. Figure 2 shows the Bland Altman plot of the tranexamic acid concentration in VAMS samples of venous and capillary blood. The average bias, lower and upper 95% limits of agreement were 0.07, −2.1 and 2.2 mg/L respectively. Figure 3 shows the Bland Altman plot of the tranexamic acid concentration in venous whole blood and VAMS capillary blood. The bias, lower and upper 95% limits of agreement were 0.7, −2.7 and 4.0 mg/L respectively. Figure 4 shows a scatter plot of the tranexamic acid concentration measured in plasma versus the corresponding concentration estimated from VAMS capillary blood samples. There was a good correlation between the values (R^2^ = 0.81, *p* < 0.001), with a Lins concordance correlation coefficient of 0.85 (95% CI 0.76–0.91). The root mean square error was 4.4 mg/L.

## Discussion

Whole blood tranexamic concentrations in samples collected using the VAMS devices correspond closely to those measured in liquid blood samples collected by venepuncture. As blood and plasma samples were stored frozen and in conditions which do not affect TXA stability (Grassin Delyle et al., 2010; Fabresse et al., 2017), the excellent agreement between TXA concentrations measured in whole blood samples and those in VAMS devices stored for the same period at ambient air suggest that VAMS storage conditions are appropriate and are not responsible for any stability issue. Our Bland Altman plots show that VAMS capillary blood samples provide reliable estimates of the TXA concentrations in venous blood and that the TXA concentrations capillary blood samples collected using VAMS correspond closely with those in blood obtained by venepuncture. The average bias of less than 1 mg/L suggests that the VAMS device is suitable for use in pharmacokinetic studies of TXA.

A major strength of our study is the use of well validated, high-sensitivity LC–MS/MS methods for the quantification of tranexamic acid concentrations. Our assay was validated in accordance with internationally recognised standards and has excellent analytical performance across the range of clinically relevant tranexamic acid concentrations in both liquid and dry samples collected using VAMS (Fabresse et al., 2017; Lamy et al., 2020). Although there was no substantial systematic differences (bias) in TXA concentrations from samples collected using the VAMS device, the intervals of agreements were wide. Whether these are acceptable is a matter for judgment that will depend on the analytic goals and practical constraints.

Pharmacodynamic studies show that plasma TXA concentrations over 10 mg/L provide near maximal inhibition of fibrinolysis, with concentrations over 5 mg/L providing some inhibition (Picetti et al., 2019). An important observation from this study is that the average TXA concentration in whole blood is approximately half the average plasma TXA concentration. We have previously shown that the distribution of TXA into red cells is almost negligible. Because TXA in the blood is almost completely contained within the plasma, the concentration of TXA measured in the plasma will necessarily be higher than in whole blood. We previously proposed that plasma TXA concentrations can be estimated from whole blood concentrations using the haematocrit and Eq. 1.

However, the “translation” of whole blood TXA levels from VAMS capillary samples into plasma levels may require caution for high concentrations. The concentration range of the present study was less than 40 mg/L, corresponding to concentrations observed in pharmacokinetic studies with an intramuscular dose of 1 g or an oral dose of 2 g (Grassin-Delyle et al., 2021a; Grassin-Delyle et al., 2021b). These pharmacokinetic studies with outpatient TXA use are precisely the types of studies for which design is most appropriate for the use of VAMS devices. However, concentrations greater than 700 mg/L may be expected with some dosing schemes used for the preventive inhibition of fibrinolysis before surgery (Grassin-Delyle et al., 2013b), and our results cannot be extrapolated to such elevated concentrations. However, although sampling of classical venous samples is not an issue with intravenous administration of high-dose TXA, the interest of VAMS sampling may be explored in such conditions due to easier analysis, storage, shipping and handling. Because our study was conducted in healthy volunteers, our results should be confirmed in patient populations. In conclusion, there was a reasonable correspondence between plasma TXA concentrations and those calculated from VAMS capillary samples, especially in the range of plasma concentrations 0–15 mg/L, which should be appropriate to properly assess the antifibrinolytic activity of TXA (Picetti et al., 2019). VAMS devices could be a means to facilitate high quality clinical research on TXA in all populations of interest.

## Data Availability

The original contributions presented in the study are included in the article/supplementary material, further inquiries can be directed to the corresponding author.
